# The impact of culturally tailored video interventions for Long COVID among Hispanic/Latino populations

**DOI:** 10.7189/jogh.16.04162

**Published:** 2026-05-15

**Authors:** Xueting Jin, Fangwu Wei, Srinivasa Srivatsav Kandala, Gilberto Lopez, Manfred D Laubichler, Tanya Bils, Rui Li, Ramesh Gorantla

**Affiliations:** 1 Decision Theater, Knowledge Enterprise, Arizona State University, Tempe, Arizona, USA; 2 School of Transborder Studies, Arizona State University, Tempe, Arizona, USA; 3 School of Complex Adaptive Systems, Arizona State University, Tempe, Arizona, USA

## Abstract

**Background:**

Long COVID has disproportionately affected Hispanic/Latino communities, particularly in Arizona. Structural inequities contribute to disparities, but limited culturally tailored health information remains an underexplored barrier to effective communication. This study evaluated the impact of culturally designed Long COVID educational videos for Hispanic/Latino audiences.

**Methods:**

We developed animated videos incorporating cultural symbols and diversity cues. Participants completed pre- and post-intervention surveys assessing Long COVID knowledge. Pre-post regression analyses examined knowledge gains, subgroup differences (education, gender, lived experience), and the influence of cultural design elements.

**Results:**

Baseline knowledge was higher among participants with greater educational attainment; however, post-intervention knowledge levels were similar across education groups. Female participants demonstrated lower scores than males, diverging from typical health information-seeking trends. Participants with lived experience of Long COVID reported higher baseline knowledge. Cultural design features, including symbolism and perceived diversity, showed modest effects, particularly among younger viewers. Overall, knowledge increased significantly following video exposure across all subgroups.

**Conclusions:**

Culturally tailored Long COVID videos effectively enhanced knowledge among Hispanic/Latino audiences, mitigating educational disparities and engaging individuals with diverse experiences. While cultural design elements had limited influence, they may enhance relevance for specific subgroups. These findings support the potential of culturally responsive video interventions to improve health education access in underserved communities and highlight areas for refinement in future multimedia health communication strategies.

Long COVID refers to a set of persistent symptoms, such as fatigue, cognitive impairment, also called brain fog, shortness of breath, and other physical or psychological issues, that continue for weeks or months after an individual recovers from an acute COVID-19 infection [1]. Between 10 and 30% of individuals who contract COVID-19 may go on to experience these long-term effects [2]. Emerging data suggest that the burden of Long COVID is not evenly distributed, with certain communities, particularly those already experiencing health disparities, reporting higher rates of ongoing symptoms [1].

In Arizona, United States, Hispanic and Latino communities have experienced a disproportionate burden of COVID-19 and its long-term consequences. Latino individuals in the state report higher rates of Long COVID symptoms than other racial or ethnic groups, with significant implications for quality of life, employment and economic stability [3]. This heightened burden reflects broader social and structural inequities, including higher poverty rates, greater exposure to frontline occupations, housing density, lower health insurance coverage, and a higher prevalence of chronic health conditions [4,5].

Among these structural inequities, an especially important and often overlooked factor is the informational gap. Hispanic and Latino populations, particularly those who are Spanish speaking, are less likely to have access to accurate, timely, and culturally appropriate health information [2,3,6,7]. Digital divides, limited trust in institutions, and the absence of culturally relevant health communication materials further deepen this gap [8–10]. As a result, individuals may delay care, misinterpret symptoms, or fail to adopt protective behaviours, not because of lack of concern but because the information they receive is inaccessible, linguistically inappropriate, or not culturally resonant.

The communication inequality framework provides a useful lens for understanding how disparities in access to, processing of, and action on health information contribute to unequal health outcomes [11]. When emerging health information, such as guidance about Long COVID, is not distributed equitably, existing disparities may widen [12]. Therefore, ensuring equitable access to health knowledge across populations is essential.

Public health education is one key strategy for reducing such disparities. Conventional approaches, including print materials, brief public service announcements, or text-heavy resources, often fail to engage diverse audiences or accommodate differences in health literacy. Video-based communication has emerged as a powerful alternative. Videos are more effective than text in enhancing message retention, illustrating abstract ideas, and appealing emotionally through storytelling and visual design [13–18]. These benefits make video particularly well suited to complex and evolving health topics such as Long COVID.

When video content is also culturally tailored, incorporating language, imagery, symbols, and narratives that reflect the lived experiences of the target population, it can be even more effective [19–21]. Theoretical models support these approaches, suggesting that personally relevant and narratively engaging messages promote deeper processing, stronger knowledge retention, and greater motivation to adopt protective behaviours [22–25]. Empirical evidence also suggests that culturally tailored messages can increase the engagement with COVID-19 preventive measures [26,27]. However, many of these studies focus on engagement metrics such as video views, likes, or shares rather than on actual knowledge acquisition. Few studies have examined how culturally tailored video interventions affect understanding across subgroups defined by age, gender, education, or lived experience with Long COVID. Differences in the ability to process, evaluate, and act on information may influence how health messages are received, making it important to understand how demographic and structural factors interact with culturally tailored interventions.

This study addresses these gaps by evaluating the impact of a culturally tailored Long COVID video campaign developed for Latino audiences in Arizona using a one-group pre-post design. Specifically, we assess:

(1) whether exposure to the videos increases knowledge about Long COVID and related perceptions,

(2) whether changes vary across demographic subgroups such as age, gender, and education, and

(3) whether and how the effects of culturally embedded elements and design features within the videos differ across population groups.

By directly measuring pre- and post-exposure differences, this study provides evidence on the effectiveness of culturally tailored video communication as a strategy to reduce informational inequities in underserved communities.

## METHODS

### Study area

This study was conducted across the state of Arizona, a region with a significant and diverse Latino population. Arizona was selected due to its high COVID-19 case burden and disproportionate impact on Latino communities, particularly in terms of long-term health outcomes [28]. Structural inequities such as health care access barriers, occupational exposure, and language divides have compounded the effects of Long COVID in this region, making it a critical area for culturally responsive public health interventions [29,30].

### Survey and animation design

To assess the effectiveness of culturally tailored video interventions on Long COVID knowledge among Latino populations, we developed a series of animated educational videos. Four animations were selected for this study. Each animation was approximately 60 seconds in length and designed to convey core information about Long COVID symptoms, prevention, and treatment.

The animation production process was deeply collaborative and culturally informed. Latino scriptwriters, cartoonists, and artists were involved throughout, ensuring that the visual narratives, character representations, and symbolic elements were culturally resonant. The creative team was first briefed with current scientific information about Long COVID, which they then adapted into culturally meaningful storylines, aesthetics, and messages designed to reflect Latino lived experiences.

This study employed a quasi-experimental one-group pretest-posttest design in which participants completed baseline measures, viewed the culturally tailored video, and then completed immediate post-test assessments. Although the absence of a control group limits the ability to draw definitive causal conclusions, the design provides important initial evidence regarding the knowledge change following exposure to the culturally tailored videos. Such approaches are widely used in early-stage communication and intervention researches to evaluate feasibility, assess preliminary effectiveness, and refine intervention components [31–33].

The survey included parallel pre- and post-animation knowledge assessments. The pretest evaluated baseline knowledge across seven core domains, including symptom recognition, risk factors, prevention through vaccination, and appropriate health care-seeking behaviours. The same items were administered after viewing the animation to measure knowledge acquisition. Additional survey items captured demographic characteristics, general health perceptions, and participant feedback regarding the cultural relevance and design effectiveness of the animation. Participants could choose either the Spanish or English version of the instrument.

### Data collection

Data was collected entirely online using the Qualtrics platform, which facilitated the recruitment of respondents through a network of validated panel partners. Recruitment targeted Latino/Hispanic adults aged 18 or older residing in Arizona. Participants were screened using eligibility criteria embedded within the survey to ensure demographic alignment with the study’s focus.

A total of 300 respondents completed the pre-post survey and animation intervention. Qualtrics implemented rigorous identity verification protocols. The survey took approximately 25 minutes to complete and included an optional follow-up qualitative interview invitation. All study procedures were approved by the Institutional Review Board at Arizona State University and adhered to best practices for online research ethics, privacy, and data security. Of the 300 completed responses, 271 respondents were retained in the final analytic sample after excluding incomplete responses and cases with missing key variables required for analysis.

### Pre-post linear regression model

This study used a pre-post linear regression model to evaluate the effectiveness of the culturally tailored video intervention on Long COVID and how such effects vary across population groups. The model included a post-intervention indicator (1 = after viewing the animation; 0 = before viewing) to capture within-person change associated with exposure. Interaction terms between the post indicator and selected covariates (*e.g*. gender and education) were included to assess whether knowledge gains differed across subgroups. The general formulation of the regression model can be expressed as follows:

*Y_it_ = β*_0_ *+ β*_1_ *Post_it_ + β*_2_ *X_i_ + β*_3_ *(Post_it_ × X_i_) + ε_it_*

where *Y_it_*  represents the knowledge score for individual i at time t (pre or post watching the animations); *Post_it_* indicates the post-intervention period, *X_i_* represents individual-level covariates, and *Post_it_ × X_i_* represents interaction terms used to assess differential knowledge change across subgroups.

Total knowledge scores were calculated by awarding one point for each correct response (range: 0–7). The seven-item knowledge scale showed moderate internal consistency (Cronbach’s α = 0.661). Ceiling effects were minimal, with 11% scoring at the maximum at baseline and 2% post-intervention, indicating adequate variability to detect change.

The independent variables come from four aspects, including sociodemographic variables, health condition of the respondents, respondent’s opinion on animation design and the interaction variables (**Table 1**).

**Table 1:** Explanation of the independent variables

Variables	Description
Post-intervention	A dummy variable
	1 = after watching the animation; 0 = otherwise
Non-White Hispanic	A dummy variable
	1 = identifies as non-White Hispanic; 0 = identifies as White Hispanic
Female × pre	Interaction term between gender and pre-intervention period
Female × post	Interaction term between gender and post-intervention period
Age	Age of the respondent
Married	Dummy variable.
	1 = married; 0 = otherwise
High education × pre	Interaction between education and pre-intervention period
High education × post	Interaction between education and post-intervention period
Self-rated good health	Dummy variable
	1 = reports good health; 0 = otherwise
Self-reported Long COVID	Dummy variable.
	1 = reports having experienced Long COVID; 0 = otherwise
Perceived diversity representation	A dummy variable
	1 = believes the animation reflects diversity within the Latino community in Arizona; 0 = otherwise
Cultural symbols × age≥60	Interaction term: perceived effective cultural symbols × age 60+
Cultural symbols × age≤34	Interaction term: perceived effective cultural symbols × age 34 or younger
Unique Latino elements × Age ≥60	Interaction term: uniquely Latino story elements × age 60+
Unique Latino elements × Age ≤34	Interaction term: uniquely Latino story elements × age 34 or younger
Liked animation × age≥60	Interaction term: liking the animation × age 60+
Liked animation × age≤34	Interaction term: liking the animation × age 34 or younger

Several sociodemographic variables are included in the model (**Table 1**) [11], highlighting disparities among social groups in their ability to access and utilise information. Previous studies consistently demonstrate that sociodemographic factors – such as race, gender, age, marital status, and education level – can influence individuals' access to information, levels of trust and preference, and patterns of service utilisation [22,34,35]. These factors may also affect how individuals acquire knowledge from culturally tailored animations about Long COVID. Among these variables, we focus particularly on gender and education level, comparing participants’ understanding of Long COVID before and after viewing the animations, to examine how knowledge acquisition differs across these groups.

Health status might also impact individuals’ knowledge acquisition related to Long COVID. Studies have shown that perceived susceptibility and personal experience with illness are key drivers of health information engagement and learning outcomes [36]. People with preexisting health conditions or a history of COVID-19 infection may be more motivated to seek health information, pay closer attention to educational content, and retain relevant knowledge due to heightened personal relevance or perceived vulnerability [37]. Conversely, individuals in good health might perceive Long COVID as less personally relevant and may engage less deeply with health messages. Therefore, varying health statuses may influence the degree to which individuals attend to, process, and retain knowledge from culturally tailored health education materials on Long COVID.

We included variables related to people’s perspective of animation design to capture aspects such as representation, cultural symbolism, and emotional resonance. By analysing these responses, we aimed to explore whether individual reactions to the animation’s design influenced the effectiveness of the intervention. Additionally, we examined the interaction between perceived animation design quality and age to explore whether the effectiveness of the design varied across age groups.

## RESULTS

### Statistical result

Overall, participants demonstrated a statistically significant improvement in Long COVID knowledge following exposure to the culturally tailored animations. Total scores were calculated for both the pre- and post-intervention assessments by awarding one point for each correct answer (**Table 2**). The descriptive analysis revealed an overall improvement in participant performance following the intervention. The mean score increased from 4.39 (standard deviation (SD) = 1.68) at baseline to 4.74 (SD = 1.29) post-intervention, indicating a positive shift in the central tendency of scores. The median score increased from 4.00 to 5.00, and the 25th percentile rose from 3.00 to 4.00, suggesting that gains were observed even among lower-performing individuals. The decrease in standard deviation indicates reduced variability in responses, and the minimum score improved from 0.00 to 1.00, further supporting the notion that participants across the performance spectrum benefited. Together, these findings indicate modest but consistent knowledge gains across the sample. A paired *t* test confirmed that the improvement was statistically significant (*t* = 3.788, *P* = 0.000186).

**Table 2:** Descriptive analysis result

Statistic	Pre_score	Post_score
Count	271	271
Mean	4.39	4.74
Standard deviation	1.68	1.29
Minimum	0	1
25th percentile (Q1)	3	4
Median (50%)	4	5
75th percentile (Q3)	6	6
Maximum	7	7

With respect to subgroup differences, gender emerged as the only demographic factor associated with post-intervention knowledge differences. Kruskal-Wallis tests were conducted to assess differences in scores across demographic groups. Results indicated no significant differences in pre-test scores by age, gender, or race/ethnicity. However, post-test scores differed significantly by gender (*P* = 0.026). Dunn’s post hoc test revealed that male participants scored significantly higher than female participants (*P* = 0.008), while no other pairwise comparisons were statistically significant. These results suggest that animated educational materials can effectively enhance understanding of long COVID-19, though differences in outcomes by gender may warrant further investigation.

### Pre-post linear regression result

**Table 3** summarises the results of the pre-post linear regression analysis, examining factors associated with participants’ understanding of Long COVID before and after viewing the culturally tailored animations. The model is statistically significant (R2 = 0.06) indicating that the included predictors accounted for a modest proportion of variance in knowledge scores, which is consistent with the multifactorial nature of individual learning outcomes in health communication contexts [38,39]. All variance inflation factors are below 5, indicating no multicollinearity concerns.

**Table 3:** Regression result

Variable	Unstandardised coefficients	*P*-value	Variance Inflation Factor (VIF)
Constant	3.745	<0.001*	
Post	0.652	0.016*	4.564
Female × pre	−0.18	0.354	2.106
Female × post	−0.414	0.033*	2.106
High education × pre	0.503	0.006*	1.563
High education × post	0.219	0.226	1.563
Self-reported long COVID	0.176	0.019*	1.018
Perceived diversity representation	0.236	0.066**†**	1.023
Unique Latino elements × age≤34	−0.421	0.059**†**	2.852
Liked animation × age≤34	0.386	0.073**†**	2.866

*Significant at 0.05 level.

†Significant at 0.1 level.

The regression results indicate that exposure to the animation was significantly associated with higher knowledge scores. The regression result showed that Post has a significantly positive impact (*P* = 0.016), indication higher score after viewing the animations. This suggests that visual, narrative-driven content can be an effective medium for public health communication, particularly when addressing complex or evolving health topics like Long COVID for Hispanic/Latino populations.

Participants with a bachelor’s degree or higher exhibited significantly greater baseline knowledge (*P* = 0.006), consistent with prior research linking education to health literacy [12,34,35]. However, since this effect is based on baseline scores, it does not suggest that the intervention was more effective for highly educated individuals – rather, it highlights existing disparities in pre-intervention knowledge.

A significant post-intervention gender interaction was observed (*P* = 0.033), indicating lower post-test scores among female participants compared with males. While prior literature often suggests that women are more proactive in seeking health information, this finding may reflect differences in content resonance, message framing, or engagement with the specific animation format.

Participants who reported having experienced Long COVID had significantly higher knowledge scores overall (*P* = 0.019), reinforcing prior work showing that personal relevance enhances health information engagement and recall.

Several animation design variables were marginally significant at the 10% level, including perceived diversity representation (*P* = 0.066), uniquely Latino elements among younger participants (*P* = 0.059), and liking the animation among younger participants (*P* = 0.073). While not below the conventional *P* < 0.05 threshold, they are interpreted as exploratory and hypothesis-generating. Such findings can still be meaningful in exploratory research, especially in behavioural contexts, to warrant further investigation in future studies with larger samples and confirmatory designs [40,41]. The results indicated that individuals who perceived the animation’s art style as reflective of Latino community diversity generally scored higher on post-viewing knowledge. This suggests that culturally resonant visuals can enhance engagement and improve the effectiveness of educational content. Although age was not directly associated with overall understanding of Long COVID, age-related differences emerged in how participants responded to the animation design elements. Younger individuals, particularly those in their 20s and 30s, showed greater knowledge gains when they reported higher engagement with the animation’s design elements. In contrast, older adults exhibited a smaller effect from animation design. This aligns with previous research suggesting that digital and multimedia formats are often more effective for younger audiences, who are typically more comfortable navigating and processing visual content [38].

## DISCUSSION

This study evaluated culturally tailored video interventions designed for Hispanic/Latino adults in Arizona and found significant increases in Long COVID knowledge following exposure. The results support the value of visual, narrative-driven media for communicating complex health information, while also revealing subgroup differences that can guide more targeted communication strategies.

Participants with higher educational attainment had greater baseline knowledge of Long COVID. This result is consistent with longstanding evidence linking formal education to higher health literacy and information-seeking behaviour, as higher education levels are often associated with greater access to health information and an enhanced ability to interpret and apply it effectively [12]. Importantly, knowledge gains after viewing the animations did not differ significantly by education level, suggesting that the videos were broadly accessible and capable of narrowing immediate information gaps. This finding highlights the potential of animated educational tools to bridge knowledge gaps and deliver accessible, comprehensible health information across diverse audiences, especially when addressing complex conditions like Long COVID that affect a wide range of populations.

A notable post-intervention gender difference was observed: female participants scored significantly lower than their male counterparts, even though women are often more proactive in health-related information seeking [42]. This result raises important questions about examine how message framing, character representation, pacing, or other design features may differentially engage men and women. Future interventions should consider incorporating participatory design with diverse gender perspectives to ensure that messaging is relevant, engaging, and inclusive.

Participants who reported prior Long COVID experience demonstrated higher knowledge overall, consistent with the idea that personal relevance is a strong motivator for health information engagement and recall [37].

While several variables related to design and cultural feedback of the animations (such as perceived diversity and cultural symbolism) approached statistical significance, they did not meet the conventional threshold (*P* < 0.05) and were thus interpreted with caution. Nevertheless, these trends point to the importance of further research into how specific cultural design elements affect viewer engagement and learning, especially among younger or more digitally oriented subgroups.

This study is not without limitations. Although common in early-stage intervention research, the absence of a control group limits causal inference and raises the possibility of testing effects. Self-reported data may introduce bias or social desirability effects. Additionally, our cross-sectional design limits causal inference, and we did not assess long-term retention or behaviour change. Future studies should include randomised controlled trial with an attention control, follow-up assessments, test behavioural outcomes (*e.g*. health care seeking or vaccination), and explore intra-ethnic variation within Latino populations.

## CONCLUSIONS

Our results support the growing body of evidence showing that culturally tailored, video-based public health interventions can effectively increase knowledge among underserved populations. At the same time, the observed subgroup differences point to the importance of disaggregating impact by gender and lived experience to ensure that such tools serve all members of a community equitably. Future campaigns should adopt a co-design approach that includes diverse voices and perspectives from the outset, helping to build not only more effective materials but also more inclusive health infrastructures.
